# Targeted and intelligent nano-drug delivery systems for colorectal cancer treatment

**DOI:** 10.3389/fbioe.2025.1582659

**Published:** 2025-04-25

**Authors:** Meihong Chai, Shihua Wang, Yuxin Chen, Xing Pei, Xueyan Zhen

**Affiliations:** ^1^ Department of Pharmacy, Xi’an Hospital of Traditional Chinese Medicine, Xi’an, Shaanxi, China; ^2^ School of Medicine, Xi’an Peihua University, Xi’an, Shaanxi, China; ^3^ Health Science Center, Xi’an Jiaotong University, Xi’an, Shaanxi, China; ^4^ Tianjin Key Laboratory of Food and Biotechnology, School of Biotechnology and Food Science, Tianjin University of Commerce, Tianjin, China; ^5^ Brigham and Women’s Hospital, Harvard Medical School, Boston, MA, United States

**Keywords:** colorectal cancer, nano-drug delivery systems, stimuli-responsive nanocarriers, oral drug delivery systems, targeted delivery, tumor microenvironment

## Abstract

Colorectal cancer (CRC) remains a highly heterogeneous malignancy with significant morbidity and mortality worldwide. Despite advancements in surgery, chemotherapy, immunotherapy, and targeted therapy, treatment efficacy is often hampered by drug resistance and systemic toxicity. In recent years, nano-drug delivery systems (NDDS) have emerged as a promising strategy to enhance therapeutic precision, reduce adverse effects, and overcome resistance in CRC treatment. This review discusses the recent advancements in NDDS for CRC treatment, focusing on the optimization of oral drug delivery systems, the development of tumor-specific targeting strategies, and the design of intelligent delivery systems responsive to the tumor microenvironment (TME). Furthermore, we summarize current challenges in NDDS translation and explore future research directions for enhancing their clinical feasibility and therapeutic impact.

## 1 Introduction

Colorectal cancer (CRC) is a highly heterogeneous malignant tumor originating in mucosal epithelial cells of the colon or rectum (Liu et al., 2024). CRC remains one of the leading causes of cancer-related mortality worldwide, with particularly high incidence rates in developed countries (Hultcrantz, 2021). Epidemiological studies have shown that CRC arises from a complex interaction between genetic susceptibility and environmental influences (Wang H. et al., 2023; Sobhani et al., 2020). The genetic factors include hereditary tumor syndromes such as familial adenomatous polyposis (FAP) and Lynch syndrome (Vieira et al., 2024; Nolano et al., 2023). Environmental factors involved high-fat and low-fiber dietary patterns, obesity, sedentary lifestyle, smoking and alcohol abuse, and other controllable risk factors (Chu et al., 2025). Of note, chronic inflammatory bowel disease (IBD), particularly ulcerative colitis and Crohn’s disease, are associated with a significantly elevated CRC risk, which may be related to genomic instability caused by the chronic inflammatory microenvironment (Adami et al., 2016; Shimomura et al., 2023).

In terms of treatment strategy, the multidisciplinary integrated treatment model (MDT) was adopted for CRC, and the choice of treatment was mainly based on the TNM stage of the tumor (Mangone et al., 2024). For localized lesions, radical surgical resection remains the preferred treatment for early CRC. Postoperative adjuvant chemotherapy significantly reduces the risk of recurrence in stage II-III patients (van Erning et al., 2013). However, the current treatment of CRC still faces many challenges. The dose-limiting toxicity of chemotherapy drugs (Zhen et al., 2023a) is common, including myelosuppression, peripheral neurotoxicity, digestive tract reaction, etc., and it is easy to produce acquired drug resistance (Yue et al., 2024; Was et al., 2022). Although targeted therapy improves efficacy, its application is limited by specific molecular markers and may cause adverse reactions such as skin toxicity, hypertension, and proteinuria (Farooqui et al., 2020). Therefore, strengthening early screening (such as fecal occult blood test, and colonoscopy), optimizing individualized treatment strategies, and developing novel targeted drugs and immunotherapy (Xiong et al., 2024a; Xiong et al., 2024b) are important directions to improve the prognosis of CRC in the future.

Due to the complexity of the biological barriers and the tumor microenvironment (Zhen et al., 2024), drugs are often limited by the low permeability at the tumor site, making it difficult for them to accumulate. At the same time, the hypoxic, acidic environment, and high levels of reactive oxygen species in the tumor microenvironment may affect the stability of the drugs. In recent years, the significant advantages of nanotechnology in cancer treatment have garnered considerable attention from researchers. The core benefits are primarily reflected in two key areas: precise targeting and intelligent controlled release (Chehelgerdi et al., 2023; Sun et al., 2023). Firstly, through surface modification techniques (such as antibodies, peptides, or specific ligands), nanomaterials can accurately identify and bind to markers overexpressed on the surface of cancer cells, thereby achieving targeted drug delivery (Di et al., 2020). This precision not only markedly enhances therapeutic efficacy, but also significantly mitigates the toxic side effects of conventional chemotherapy drugs on healthy tissues. Secondly, by engineering carrier materials with environmental responsiveness (such as pH-sensitive, temperature-sensitive, or enzyme-sensitive materials), the nano-delivery system can achieve controlled drug release in response to specific stimuli within the tumor microenvironment. This intelligent release mechanism prolongs drug action time, reduces dosing frequency, and improves patient compliance (Li X. et al., 2023; Wang et al., 2021).

Nano-drug delivery systems (NDDS) represent an advanced class of nanomaterials, derived from either natural or synthetic polymers (Safari and Zarnegar, 2014). These systems encompass solid particles and gel-like structures. By integrating anticancer agents with nano-delivery platforms, various functional formulations such as liposomes, polymer microspheres, micelles, and *in situ* gels can be developed (Sandha et al., 2025; Ni et al., 2020; Zhang et al., 2023b; Si et al., 2021). These nanotherapeutics significantly enhance drug targeting and bioavailability while effectively mitigating off-target effects that often lead to damage in normal tissues, a common issue with traditional small-molecule anticancer drugs (Chuang et al., 2022). With the rapid advancement of nanotechnology, its application in cancer theranostics has expanded considerably, leading to breakthroughs in treatment efficacy and demonstrating substantial potential for early diagnosis and real-time monitoring, thereby offering new hope and improved survival prospects for cancer patients (Bano et al., 2023; Seyfoori et al., 2023).

In this review, we systematically delineate the advancements in NDDS for CRC treatment, with a focus on three pivotal innovative directions: the optimization of oral drug delivery systems, the development of tumor-specific targeting strategies, and the design of tumor microenvironment-responsive intelligent delivery systems. Through a comprehensive analysis of the groundbreaking progress achieved by nanocarriers in enhancing oral drug bioavailability, improving tumor-targeting specificity, and enabling microenvironment-regulated drug release, we aim to provide novel insights and a robust theoretical foundation for the development of next-generation intelligent NDDS. The integration of these cutting-edge nanotechnologies is anticipated to significantly enhance the precision and efficacy of CRC treatment. By improving therapeutic outcomes and reducing the risk of recurrence, these advancements not only promise to elevate the treatment experience and quality of life for patients, but also hold the potential to extend overall survival rates. Collectively, these innovations pave the way for transformative approaches in the clinical management of CRC.

## 2 Recent advancements in NDDS for CRC treatment

### 2.1 Oral drug delivery systems

**FIGURE 1:**
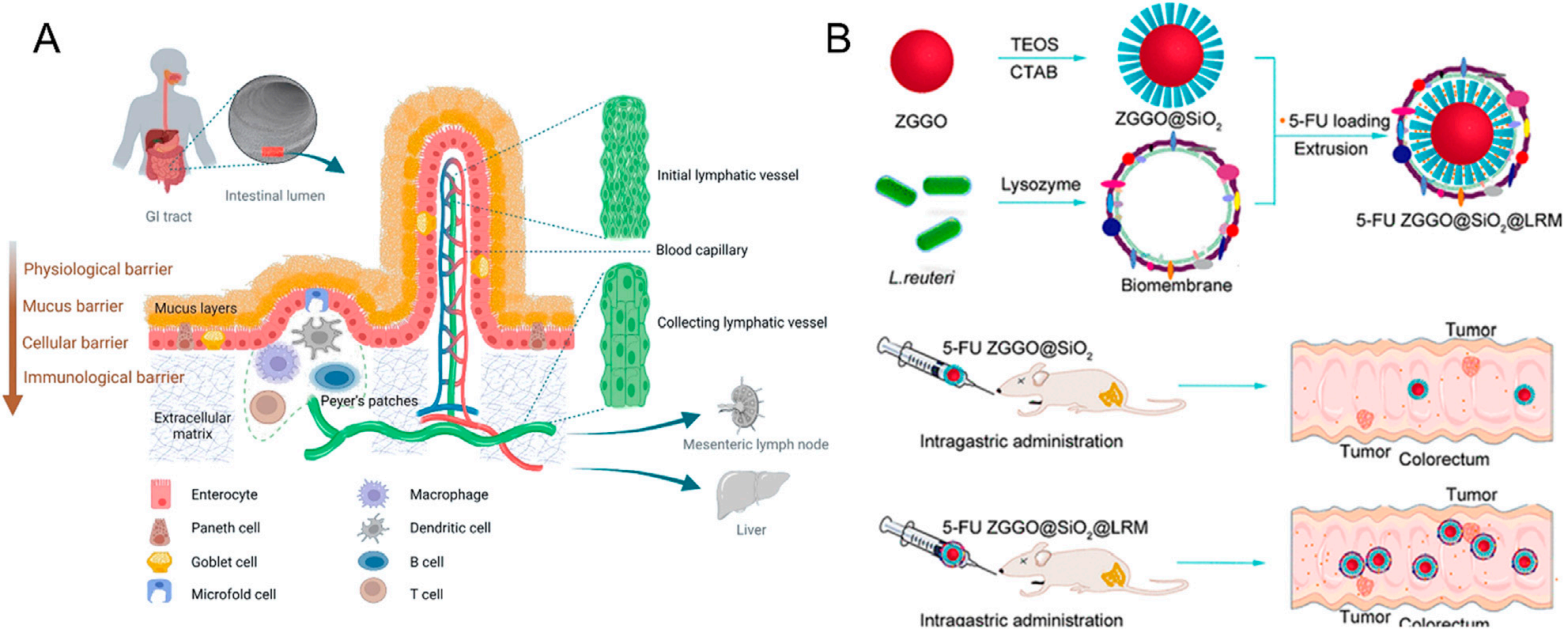
**(A)** Schematic diagram based on the transit pathway of nanoparticles in the gastrointestinal tract after oral administration (Reproduced with permission from the reference: Mechanisms of Nanoparticle Transport across Intestinal Tissue: An Oral Delivery Perspective). Adapted from Ejazi et al., ACS Nano, 2023, under a Creative Commons CC BY license. **(B)** Schematic Diagram of 5-Fu ZGGO@SiO_2_@LRM Nanoparticles for Colorectal Cancer Imaging and Chemotherapy (Reproduced with permission from the reference: Bacterial Biofilm Bioinspired Persistent Luminescence Nanoparticles with Gut-Oriented Drug Delivery for Colorectal Cancer Imaging and Chemotherapy). Adapted from Wang et al., ACS Applied Materials and Interfaces, 2019, under a Creative Commons CC BY license.

Oral administration is a preferred route for drug delivery due to its non-invasiveness, patient compliance, and suitability for both local and systemic therapies (Liu et al., 1997). This mode of drug delivery utilizes the extensive mucosal absorption surface area of the intestine and prolonged drug retention time, making it an ideal choice for the management of gastrointestinal diseases (Chai et al., 2025). Its non-invasive nature, convenience, and high patient compliance further underscore its clinical appeal (Chen et al., 2023). However, traditional oral drugs face significant challenges in the complex microenvironment of the digestive tract, which can severely compromise their efficacy (Ejazi et al., 2023) (Figure 1A). Factors such as the highly acidic gastric environment (pH 1.0–3.0), enzymatic degradation by gastrointestinal digestive enzymes, and the barrier properties of the gastrointestinal mucosa insignificantly reduce drug bioavailability (Bashiardes and Christodoulou, 2024). To address these limitations, the development of novel intelligent oral NDDS with environment-responsive characteristics has emerged as a critical scientific challenge in the field of drug delivery. These advanced systems are designed to effectively protect drug activity and enhance targeted delivery efficiency. The successful development of such innovative delivery platforms is anticipated to not only significantly improve drug bioavailability, but also provide transformative solutions for the precision treatment of gastrointestinal diseases.

#### 2.1.1 NDDS developed based on low stomach pH

There were significant pH gradients in different anatomical parts of the digestive tract. Physiologically, the stomach cavity maintains a strongly acidic environment (pH 1.0–3.0), while as chyme advances into the distal digestive tract, the pH of the duodenal-ileum segment gradually increases to the weakly alkaline range (pH 6.0–7.4) (Collnot et al., 2012; Koziolek et al., 2015). Notably, the CRC tissue microenvironment is typically weakly acidic (pH 6.5–7.2) (Gerweck and Seetharaman, 1996). Based on this unique pH distribution in the digestive tract, researchers have successfully developed a variety of PH-responsive smart delivery materials, including polymethacrylate polymers, natural polysaccharides chitosan, and synthetic polymers such as Eudragit (Turanlı and Acartürk, 2022; Noreen et al., 2024; Gupta et al., 2023). These functional materials can undergo controlled physicochemical changes such as swelling, dissolution, or degradation at specific pH thresholds, thereby achieving precise controlled release of therapeutic drugs at target sites, improving drug bioavailability, and reducing systemic toxicity (Wathoni et al., 2020).

Bajracharya et al. developed an innovative colon-targeted drug delivery system using methotrexate (MTX) as a model drug. The system was constructed by decorating gold nanoparticles onto aminoclay (AC-Au) and coating them with pH-sensitive methacrylic acid-co-methyl methacrylate (1:2) copolymer. Experimental results demonstrated that the functionalized nanoparticles exhibited excellent pH-dependent release characteristics: In the simulated gastric environment (pH 1.2), only approximately 23% of the drug was released, while in the simulated colonic environment (pH 7.4), the drug release rate reached 75%, which was attributed to the pH-responsive dissolution of the outer polymeric coating (Bajracharya et al., 2023). This novel pH-responsive delivery system significantly enhances the targeting efficiency and *in vivo* antitumor efficacy of MTX in CRC treatment.

Wang and his team pioneered the design and synthesis of deoxycholic acid and hydroxybutyl decorated chitosan nanoparticles (DAHBC NPs), establishing a groundbreaking oral drug delivery system aimed at enhancing the bioavailability of the poorly soluble drug curcumin (CUR) (Wang T. et al., 2019). These nanocarriers exhibit a distinctive temperature-responsive behavior, characterized by a low critical solution temperature (LCST) ranging from 27°C to 33°C, which is notably below physiological temperature (37°C). In simulated gastric conditions (pH 1.2, 37°C), DAHBC NPs demonstrate remarkable stability, primarily attributed to the dynamic equilibrium between pH-responsive expansion and thermal-responsive contraction. Upon transitioning to simulated intestinal conditions (pH 7.0–7.4, 37°C), the thermal-responsive contraction properties of DAHBC NPs become predominant, facilitating the rapid release of CUR. Experimental findings reveal that this delivery system effectively modulates CUR release in the stomach, with a release rate of merely approximately 10%. Notably, DAHBC NPs enhance the intestinal absorption efficiency and bioavailability of CUR by approximately tenfold compared to free CUR, underscoring the immense potential of this nano-delivery system in augmenting the oral absorption of insoluble drugs.

Eudragit, a pH-sensitive enteric polymer, demonstrates excellent solubility at pH 5.5 due to its unique solubility characteristics (Kendall et al., 2009). This polymer exhibits superior drug retention capabilities compared to other similar polymers, offering significant advantages in pharmaceutical applications. Its distinctive properties not only effectively shield active pharmaceutical ingredients from degradation in the acidic gastric environment, thereby enhancing drug bioavailability, but also enable precise drug release at targeted intestinal sites (Nikam et al., 2023). In a groundbreaking study, the Kassem developed an innovative drug delivery system by utilizing Eudragit®-S100 (EUS-100) for surface modification of functionalized silica samples (Kassem, et al., 2024). Their research demonstrated that the EUS-100 coating effectively prevents premature release of encapsulated catechins (CHT) in the acidic gastrointestinal environment while maintaining the chemical stability of catechins against pH variations. Notably, the experimental results revealed that approximately 90% of catechins were specifically released at the colon site, highlighting the crucial role and application potential of Eudragit in colon-targeted drug delivery systems.

In addition, bionics has demonstrated significant application potential in the development of functional nanomaterials (Tang et al., 2024). For instance, certain acid-tolerant bacteria can survive the harsh stomach environment and colonize the gut, offering valuable insights for the design of nanomedicine delivery systems (Li X. et al., 2025). Leveraging this bionic principle, Wang and his research team innovatively coated *Lactobacillus reuteri* biofilm (LRM) onto the surface of near-infrared persistent luminescent mesoporous zinc gallogermanate (ZGGO), successfully constructing nanoparticles with bacterial bionic properties (ZGGO@SiO_2_@LRM) (Wang Z.-H. et al., 2019) (Figure 1B). This nano-system not only withstands the acidic stomach environment, but also achieves targeted delivery of 5-fluorouracil (5-FU) to the colorectal region. Experimental results revealed that the LRM coating significantly enhanced the colonization of nanomaterials in the colorectal area and markedly prolonged the retention time of drugs at the target site. *In vivo* tracking demonstrated that the nanomaterial could still be detected in the colorectal region 24 h post-administration. This groundbreaking study provides novel insights and a theoretical foundation for the development of oral targeted colorectal drug delivery systems, holding substantial promise for clinical applications.

#### 2.1.2 NDDS developed based on different enzyme systems in the gastrointestinal tract

The gastrointestinal tract harbors an intricate enzymatic network that functions as highly specialized molecular shears, cleaving chemical bonds in most pharmaceutical compounds and rendering them biologically inactive prior to reaching their target sites (Udenigwe et al., 2021). To circumvent this enzymatic degradation and ensure site-specific drug delivery, polysaccharides including chitosan, guar gum, and pectin are extensively employed as rate-controlling excipients in colon-targeted drug formulations (Shahdadi Sardou et al., 2019; Shao et al., 2025; Udaipuria and Bhattacharya, 2025). These polysaccharide-based matrices demonstrate remarkable resistance to gastric and intestinal enzymatic activity, maintaining structural integrity throughout the upper gastrointestinal tract. However, upon reaching the colonic environment, they undergo selective degradation by anaerobic bacterial flora, thereby initiating controlled drug release (Salyers et al., 1978). This sophisticated drug delivery strategy not only enhances site-specificity, but also optimizes therapeutic efficacy through targeted pharmacological action.

**FIGURE 2:**
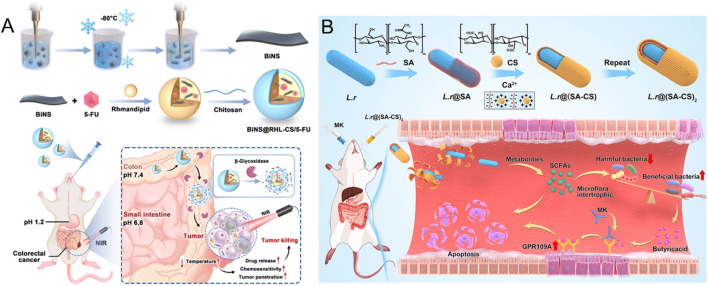
**(A)** The mode and mechanism of action of BiNS@RHL-CS/5-FU NPs (Reproduced with permission from the reference: β-Glycosidase sensitive oral nanoparticles for combined photothermal and chemo treatment of colorectal cancer). Adapted from Zhou et al., Journal of Materials Chemistry B, 2024, under a Creative Commons CC BY license. **(B)** Schematic illustration of L.r @(SA-CS)_2_ suppressed tumor growth by modulating intestinal flora (Reproduced with permission from the reference: Taking SCFAs produced by Lactobacillus reuteri orally reshapes gut microbiota and elicits antitumor responses). Adapted from Li et al., Journal of Nanobiotechnology, 2024, under a Creative Commons CC BY license.

Chitosan is a linear polysaccharide formed by randomly distributed N-acetyl-D-glucosamine and D-glucosamine units connected by β (1–4) glucoside bonds (Zamboulis et al., 2020). Its unique molecular structure enables it to be degraded by specific enzymes secreted by the colon microbial community, which makes chitosan a significant application in the targeted therapy of CRC (Ray, 2019). In an innovative study by Zhou et al., the research team successfully developed a novel oral drug delivery system (Zhou et al., 2024) (Figure 2A). The system uses rhamnolipid (RHL) to encapsulate the chemotherapy drug 5-FU and the photothermic agent bismuth nanosheets (BiNS), and chitosan (CS) is used as the shell of the nanoparticles. The BiNS@RHL-CS/5-FU nanoparticles (NPs) were constructed. The delivery system has unique pH response characteristics: During gastrointestinal transport, the double protective barrier formed by rhamnose-lipids and chitosan can effectively prevent the premature release of BiNS and 5-FU. When the nanoparticles reach the colonic site, β-glycosidase can specifically degrade the chitosan shell under the condition of elevated microenvironmental pH. This process not only reduces the size of nanoparticles, enhances their penetration and absorption ability in tumor tissues, but also significantly improves the tumor targeting efficiency of 5-FU, and effectively reduces the toxic effect of the drug on normal tissues.

Tramontano et al. found that although gelatine-coated diatomite nanoparticles (diatomite NPs) show potential applications in drug encapsulation and cell delivery, their oral administration effects are significantly limited (Tramontano et al., 2022). This is mainly due to the degradation of gelatin by digestive enzymes in the gastrointestinal tract, resulting in premature release of the active ingredient before the target site. To solve this problem, the researchers innovatively adopted microfluidic nanoprecipitation technology to further encapsulate NPs in hydroxypropyl methylcellulose acetate succinate (HPMC). This enteric polymer not only effectively protects the gelatin coating on the surface of the NPs from gastric enzymatic hydrolysis, but also enables the precise release of the drug in the specific pH environment of the intestine, thus significantly improving its therapeutic effect on colon cancer.

In fact, very few unmodified proteins survive in the stomach and make it to the intestines. Pepsin is activated at low pH and efficiently hydrolyzes peptide bonds in proteins, breaking them down into smaller peptide fragments (Rivera del Rio et al., 2021). As a result, unprotected proteins are often quickly digested in the stomach, making it difficult to maintain their integrity and biological activity. However, binding with specific carrier molecules or encapsulation in acid-resistant NPs is an important strategy to avoid the inactivation of protein drugs during oral administration. In the study of Elmorshedy et al., they developed an NDDS based on this idea, and the construction of the system adopted advanced nanotechnology: First, two active molecules docetaxel (DTX) and atorvastatin (ATR) were loaded onto lactoferrin (Lf) nanocapsules to form stable nanoparticles through chemical conjugations; Subsequently, the molecular interaction between carboxymethyl inulin (CMI) or carboxymethyl cellulose (CMC) and zeololysin (ZN) was utilized to further microencapsulated the nanoparticles and construct colon-targeting NDDS (Elmorshedy et al., 2023). *In vitro* release experiments showed that the delivery system showed good stability in the simulated gastric fluid environment, and no drug leakage was detected. In the colon environment, the nanoparticles were released efficiently and showed significant cancer cell targeting and excellent anti-tumor effect.

#### 2.1.3 NDDS developed based on the dense mucus barrier of the gastrointestinal tract

In addition to the well-known extreme pH and complex enzymolysis barriers, the unique physiological characteristics of gastrointestinal mucosal tissue constitute another important obstacle. Specifically, the surface of the gastrointestinal tract is covered by a mucous layer about 50–450 μm thick, with a pore size of about 50–1800 nm and a negative charge, forming a complex physicochemical barrier (das Neves et al., 2020; Damianos et al., 2025). Drug molecules must first overcome the electrostatic rejection and steric hindrance of the mucus layer, and interact with mucin specifically or non-specifically in order to achieve effective penetration, and then reach the epithelial cells to play a role (Subramanian et al., 2022). In recent years, the breakthrough of nanotechnology in the field of drug delivery has provided a new way to solve this problem. Studies have shown that nanoparticle delivery systems in the size range of 50–200 nm exhibit excellent mucus penetration (Öztürk et al., 2024). Currently, biomaterials commonly used to improve mucus permeability include: chitosan (cationic properties and bioadhesion), hyaluronic acid (good biocompatibility and mucus affinity), alginate (excellent gel formation ability), and other polysaccharide substances with specific functional groups (Lu et al., 2020; Zhang, Y. et al., 2023a; Taipaleenmäki et al., 2020).

Chitosan can enhance the transepithelial permeability of anticancer drugs by temporarily opening tight junctions between epithelial cells (Cole et al., 2018). Wang et al. developed an innovative hydrogel delivery system based on methacrylic anhydride-modified carboxymethyl chitosan (MA-CMCS) (Wang C.-Y. et al., 2022). The system successfully loaded and delivered imatinib (IMT) by combining the osmotic accelerator sodium deoxycholate (SD). This delivery system not only has enzyme-responsive long-acting drug release properties, but also synchronously regulates epithelial tight junctions and significantly improves intestinal permeability. This innovative research provides a new strategy and theoretical basis for improving the oral bioavailability of hydrophobic anticancer chemotherapy drugs.

The carboxyl group on the alginate molecular chain can electrostatically interact with the positively charged protein in the colon mucosa to form a hydrophilic adsorption layer (Szekalska et al., 2023). Alginate can form gel-protected loaded active molecules in an acidic environment and is specifically degraded under the action of reduction to solution in the colonic environment, such as glucuronidase, which makes it an ideal colon-targeting carrier material (Lee et al., 2025). Studies have shown that the mucosal adhesion properties of alginate can significantly prolong the retention time of the drug at the absorption site, thereby effectively improving the bioavailability of the drug (Kolawole et al., 2023). In recent years, researchers have made breakthroughs in the development of alginate-based delivery systems. The Abbasi research team has innovatively designed a folate-functionalized alginate nanoparticle-hydrogel composite delivery system for carrying the hydrophobic anti-tumor drug diferuloylmethane (Abbasi et al., 2023). The delivery system skillfully integrates the advantages of a high drug load of nanoparticles, the slow-release property of hydrogel, and the active targeting function of the folic acid receptor, successfully overcoming the key problem of low bioavailability of diferuloylmethane in the treatment of CRC, and provides a new technical strategy for improving its therapeutic effect. In addition, Li and other researchers adopted advanced layer-by-layer assembly technology (Li et al., 2024) (Figure 2B). A probiotic microgel delivery system ((L.r@ (SA-CS)_2_) was constructed based on sodium alginate (SA) and chitosan (CS). This system not only significantly improves the survival rate of *Lactobacillus reuteri* (L.r) in the harsh gastrointestinal environment, but also achieves the controlled release of probiotics in the gut by precisely regulating the swelling and degradation behavior of the hydrogel. *In vitro* and *in vivo* experiments confirmed that the delivery system can effectively regulate the intestinal microecological balance, and showed a significant synergistic effect in anti-tumor therapy, providing a new idea for tumor microecological therapy.

### 2.2 Targeted NDDS

In recent years, NDDS, as an emerging therapeutic strategy, has shown great potential in tumor-targeted therapy (Batool et al., 2023). Excellent targeted delivery capabilities enable the precise delivery of drugs packaged in nanocarriers to the site of action, which can enhance therapeutic effectiveness and reduce side effects. The application of nanomedicine delivery systems in the treatment of CRC primarily employs two strategies: passive targeting and active targeting.

#### 2.2.1 Passive targeting

It is generally accepted that solid tumors are usually highly heterogeneous, with significant differences in the vascular system depending on tumor type and stage of growth (Pe’er et al., 2021). It is worth noting that tumor tissues generally lack functional lymphatic vessels and there is a large gap between endothelial cells, which leads to the easy exosmosis or retention of macromolecules. This unique pathophysiological property promotes the selective accumulation of nanoparticles in tumor tissues, a phenomenon defined as enhanced permeability and retention (EPR) (Maeda et al., 2000). The passive targeting strategy based on the EPR effect can significantly improve the specific distribution of drugs in tumor tissues and minimize the toxic effect on normal tissues through the rational design of nanocarriers (such as liposomes, polymer nanoparticles, etc.).

Yuan et al. adopted silk fibroin (SF) as the carrier material of the drug delivery system, and successfully constructed a dual-drug co-loaded hydrogel system (Yuan et al., 2025). The team combined 5-FU in SF solution with curcumin dissolved in acetone solvent through precise process control to produce a nanoparticle drug delivery system with a particle size distribution ranging from 77.87 nm to 299.22 nm. It is worth noting that this composite nanosphere exhibits a significant EPR effect, which enables it to effectively enrich solid tumor tissue through a passive targeting mechanism, thus enhancing tumor therapy.

**FIGURE 3:**
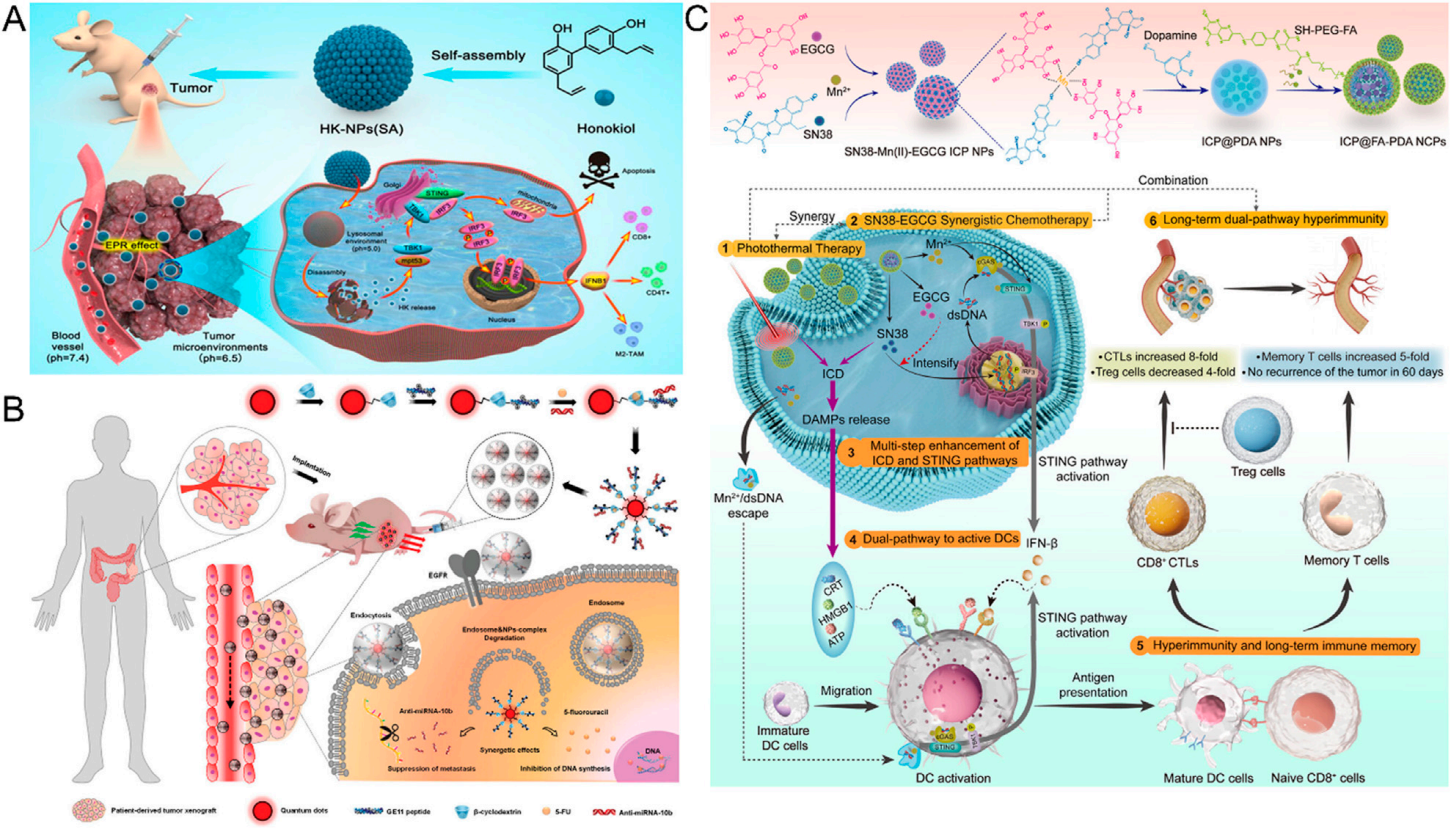
**(A)** With the EPR effect, the accumulation of HK NPs at the tumor site for the treatment of colorectal cancer (Reproduced with permission from the reference: Natural Small Molecules Enabled Efficient Immunotherapy through Supramolecular Self-Assembly in P53-Mutated Colorectal Cancer). Adapted from Ji et al., ACS Applied Materials and Interfaces, 2022, under a Creative Commons CC BY license. **(B)** Mechanism of GCD-targeted therapy in EGFR-overexpressing colorectal cancer (Reproduced with permission from the reference: Enhancing the Management of Metastatic Tumors by Robust Co-Delivery of 5-Fluorouracil/MicroRNA-10b Inhibitor Using EGFR-Targeted Nanovehicles). Adapted from Wang et al., Advanced Healthcare Materials, 2023, under a Creative Commons CC BY license. **(C)** Schematic synthesis and antitumor mechanism of ICP@FA-PDA (Reproduced with permission from the reference: Infinite Coordination Polymer Polydopamine Nanocomposites Dual-Pathway Multistep Induction of Long-Term Hyperimmunity Combined with Photothermal-Chemo Synergistic Therapy Colorectal Cancer). Adapted from Zhang et al., Aggregate, 2025, under a Creative Commons CC BY license.

In view of the complex variability and potential toxicity of nanocarriers, Ji and other researchers have carried out innovative research based on natural small molecules (Ji et al., 2022) (Figure 3A). They found that honokiol (HK) can self-assemble through intermolecular hydrogen bonds and hydrophobic interactions to form stable NPs. This self-assembled system based on natural ingredients exhibits unique tumor-targeting properties: First, HK NPs can selectively accumulate at the tumor site thanks to the EPR effect; Subsequently, they enter the cell through endocytosis of tumor cells. Finally, under the weakly acidic condition characteristic of the TME, HK molecules are released from the NPs in a controlled manner, thereby maintaining a sustained drug concentration within the tumor cells and exerting its cytotoxic effects.

However, the limitation of passive targeting is that its targeting is relatively weak, and it is greatly affected by tumor heterogeneity and individual differences (Ojha et al., 2017). Therefore, it is necessary to combine EPR-based NDDS with other mechanisms to enhance their targeting.

#### 2.2.2 Active targeting

Active targeting, by contrast, modifies specific targeting molecules (such as antibodies, peptides, aptamers, etc.) on the surface of the nanocarriers so that they can accurately recognize and bind to specific antigens or receptors on the surface of tumor cells, thus achieving efficient drug delivery. Active targeting strategy not only significantly improves the targeting and therapeutic effect of drugs, but also further reduces systemic toxicity, providing a new idea for the precise treatment of CRC. In recent years, the design concept of a nano-drug-targeted delivery system for CRC is mainly based on the “ligand-receptor” specific binding principle (Xu et al., 2020). By screening specific receptors that are highly expressed in CRC cells and selecting the corresponding ligands for surface modification of the nanoparticles, the researchers realized the active targeted therapy of the drug on the tumor cells. Among many highly expressed tumor-related receptors, CD44, epidermal growth factor receptor (EGFR), folate receptor (FR) and transferrin receptor (TfR) are the most widely studied targets (Rao et al., 2013; Napolitano et al., 2024; Zhen et al., 2023b; Soe et al., 2019; Kim et al., 2023).

As a key regulatory molecule in tumor progression and metastasis, overexpression of CD44 has been shown to be significantly associated with poor prognosis in patients with rectal cancer (Du et al., 2008). In view of the important regulatory role of CD44 in tumor-targeted therapy, researchers have developed a variety of nano-delivery systems targeting CD44. Among them, Gonzalez-Valdivieso et al. innovatively designed a multifunctional nanohybrid system that integrates Akt inhibitors with DTX through chemical coupling and specifically binds to CD44 DNA aptamers (Gonzalez-Valdivieso et al., 2023). The experimental results showed that the nano-system had a significant selective inhibitory effect on CRC cells, reducing cell survival to 2.5%. It is worth noting that the specific recognition ability of hyaluronic acid (HA) and CD44 on the surface of tumor cells provides an important way to achieve active targeting. On this basis, Phatak et al. developed a novel dual-polymer coating system, which encapsulated sorafenib in an HA-modified poly-lactic-co-glycolic acid (PLGA) - polysarcosine (PSAR) dual-polymer shell (Phatak et al., 2023). The specific delivery of the drug to CD44 overexpressed CRC cells was successfully achieved. In addition, Hu et al. built NDDS by integrating HA with pheophorbide A (PPa) and NLG919 heterodimers (Hu et al., 2020). This system not only makes full use of the active targeting properties of HA, but also realizes the precise release of drugs at the target through the cleavage of NSP disulfide bond triggered by glutathione in the tumor microenvironment and HA degradation mediated by hyaluronidase. The introduction of this dual response mechanism significantly improves the tumor targeting and therapeutic efficacy of drugs, providing a new strategy for targeted therapy of CRC.

Activation of the EGFR and its downstream signaling pathway plays a key role in the onset and progression of CRC (Catalano and Trusolino, 2019). Monoclonal antibodies (MoAbs) targeting EGFR, such as cetuximab and panizumab, have made significant progress in the treatment of metastatic CRC (mCRC) (Stroh et al., 2010). To enhance the therapeutic effect, the researchers developed anti-EGFR antibodies or ligand-modified nanoparticles that are able to specifically target cancer cells with high EGFR expression. For example, Fang and his team synthesized mixed NPs “Cet-CINPs” and coupled their surfaces to cetuximab to achieve precise targeting of tumors (Fang et al., 2023). In addition, by introducing a photosensitizer into the NPs, the NDDS showed enhanced cell uptake and significant cytotoxic activity under near-infrared light irradiation, thus combining targeted therapy with chemical photothermal therapy and providing a new strategy for CRC treatment. In 2023, Wang et al. developed a nanocarrier called GCD (Wang D. et al., 2023) (Figure 3B). The vector is composed of GE11 peptide, bioprobe quantum dots (QD), and β-cyclodextrin (β-CD), which has EGFR targeting properties. GCD nanocarriers can simultaneously deliver different targets of 5-FU and anti-Mir-10b to CRC, especially in anti-CRC metastasis, showing potential application value. This study provides new ideas and tools for the precise treatment of CRC.

FR, as a target that is overexpressed on the surface of a variety of cancer cells but underexpressed in normal cells, has attracted much attention in the development of tumor-targeting nano-drug delivery systems (Frigerio et al., 2019). The specific binding of the folate-drug complex to the folate receptor can not only enhance the retention of the drug at the target site, but also effectively reduce the drug resistance of colon cancer cells (Mallik et al., 2024). Based on this principle, Ge et al. innovatively designed a F2-C-5-FU-FA constructed by the covalent connection of *Angelica gigas* polysaccharide (F2), carboxymethyl-5-fluorouracil (C-5-FU) and FA (Ge et al., 2024). The results of *in vivo* and *in vitro* experiments showed that the nano-conjugated compound showed excellent selectivity to cancer cells with high expression of folic acid receptor, and significantly reduced the non-specific toxicity of the drug to immune organs. Similarly, the Ma research team used the same strategy to prepare CFP2-C-5-FU-FA NPs, and confirmed through *in vivo* fluorescence imaging technology that the fluorescence signal intensity of the targeted nanoparticles in the tumor region was significantly higher than that of the non-targeted control group, and had lower cardiac toxicity (Ma et al., 2023). Zhang et al. developed a folate-modified polydopamine (PDA) infinite coordination polymer nanocomposite (ICP@FA-PDA) (Zhang X. et al., 2025) (Figure 3C). The system not only realizes the efficient utilization of drug components, but also has the ability of strong immune response induced by dual pathways and precise targeting mediated by folic acid. *In vitro* experimental data showed that the nanocomposite material had a 100% inhibition rate on primary tumors in mice, and no tumor recurrence was observed during the observation period of 60 days. In recent years, magnetic nanoparticles with superparamagnetic properties have been widely used in the biomedical field because of their ability to be regulated by external magnetic fields. Under this research background, Alnasraui et al. successfully designed and synthesized Fe_3_O_4_@Au-DEX-CP-FA multifunctional nanoparticles (Alnasraui et al., 2023). The system adopts the structure design of a gold shell-coated ferric oxide core, which not only enhances the stability of the nanoparticles, but also prevents the oxidation of the core. They integrated dexamethasone (DEX) and cyclophosphamide (CP) as active molecules into the nano-system, and functionalized folic acid in the outermost layer, thus achieving precise targeting of tumor sites.

Transferrin receptor (TfR) is highly expressed on the surface of various cancer cells, which provides a theoretical basis for TfR-mediated targeted drug delivery by Tf-modified nanoparticles (Trabolsi et al., 2022). In a related study, Cheng et al. innovationally used genetic engineering technology to construct recombinant mouse heavy chain ferritin (mHFn), which not only has a high affinity for TfR, but also contains a unique heat-sensitive channel structure (Cheng et al., 2024). Mitoxantrone (MTO) was successfully encapsulated in the internal cavity of mHFn (mHFn@MTO). Under the irradiation of 660 nm laser, the photothermal effect of MTO can rapidly increase the local temperature and trigger the opening of heat-sensitive channels, thus achieving rapid release of MTO in a heat-responsive manner and significantly inducing apoptosis of tumor cells. In addition, Wei et al. developed a transferrin binding peptide (TBP) functionalized polymer carrier system that specifically binds Tf and mediates the targeted delivery of doxorubicin (Dox) to TfR-overexpressed CRC cells (Wei et al., 2020). The experimental results showed that, compared with common polymer-doxorubicin complex (Ps-Dox), Tf@TBP-Ps-Dox had a longer blood circulation half-life and significantly enhanced tumor inhibition effect on HCT-116. It is worth noting that nanoparticles are easy to form protein corona (PC) in the biological fluid environment, and this surface-adsorbed biomolecular layer may mask the targeting ligand, resulting in loss of targeting function. To address this challenge, Zhang et al. designed a Tf functionalized superparamagnetic iron oxide nanoparticle (SPIO-Tf) based on remote magnetothermal stimulation, which was modified by polyethylene glycol and grafted with Tf on the surface (Zhang T. et al., 2025). It was found that after the application of alternating magnetic field (AMF) stimulation, the local heat generated by SPIO-Tf can induce PC recombination, so that the shielded Tf can be re-exposed to the surface of the nanoparticles, thus significantly improving its tumor-targeting efficiency.

In addition, mannose receptors (MR) and EpCAM (epithelial cell adhesion molecules) are also effective targets for CRC treatment (Dalle Vedove et al., 2018; Gires and Bauerle, 2010). Bai et al. constructed a new nanomedicine delivery system, RG@M- γ-CD CNPs (Bai et al., 2021). The core structure of the system is γ-cyclodextrin (γ-CD), and its hydrophobic cavity can effectively contain the antitumor drug regorafenib (RG). At the same time, mannose modification in the outer layer gives the nanosystem the ability to specifically target tumor cells. Ge et al. developed a bioconjugate consisting of EpCAM aptamers, polyethylene glycol, and dendrimers for specific delivery of celastrol to EPCAM-rich tumors to improve anti-tumor efficacy and mitigate toxicity (Ge et al., 2020). Due to the modification of EpCAM aptamer, the NDDS can actively target tumors, which greatly reduces the acute toxicity of celastrol. Since the particle size is less than 40 nm and has a high negative surface charge, it can efficiently penetrate the tumor upon reaching the tumor site.

The surface of NDDS can be conjugated with targeting moieties such as antibodies or ligands to specifically bind overexpressed receptors on target cell surfaces, thereby facilitating drug internalization and avoiding off-target effects. Different receptor-targeted NDDS exhibit unique advantages and limitations in CRC treatment, with performance disparities primarily stemming from the complexity of target biology, carrier design, and TME interactions.

CD44, as a hyaluronic acid receptor highly expressed on CRC stem cells, enables hyaluronic acid-based nanocarriers to effectively penetrate the tumor extracellular matrix and eliminate cancer stem cells, reducing recurrence risk (Wang K. et al., 2022). However, its widespread expression in normal tissues may induce off-target toxicity, while CD44 isoform heterogeneity limits targeting precision (Cisterna et al., 2016). EGFR-targeted systems achieve efficient drug delivery via receptor-mediated endocytosis, demonstrating significant efficacy in EGFR-overexpressing wild-type tumors. Nevertheless, drug resistance in KRAS-mutated CRC and EGFR signaling-induced immunosuppressive TME restrict therapeutic outcomes (Parseghian et al., 2019; Woolston et al., 2019). FR-targeted systems leverage the high-affinity interaction between folate and FR-α to precisely recognize FR-positive tumor cells, with the potential to cross the blood-brain barrier (BBB) for brain metastasis treatment. However, competition with plasma folate may reduce tumor accumulation (Wang K. et al., 2022). TfR-targeted systems selectively deliver drugs to highly proliferative tumor cells via iron-dependent receptor-mediated endocytosis, however, TfR expression in normal tissue cells limits safety (Bajracharya et al., 2022).

Collectively, these systems face challenges including TME heterogeneity, immune clearance, and scalability. Future development requires integrating multitargeted strategies to overcome heterogeneity, designing smart responsive carriers for TME-specific drug release, and optimizing patient stratification via personalized medicine. Despite technical bottlenecks, receptor-targeted nano-systems represent a promising innovation in CRC therapy, with clinical translation dependent on deep interdisciplinary integration of materials science, molecular biology, and clinical research.

### 2.3 NDDS based on TME

TME is a complex ecosystem composed of malignant and non-malignant cells, an extracellular matrix, and various biomolecules (Li Y. et al., 2022), playing a crucial role in tumor progression and therapy resistance. TME is not only a key driver of tumor progression but also an important basis for tumor resistance therapy and immune escape (Xiao and Yu, 2021). Typical features of TME include moderate acidity (pH 6.5–7.0), excessive secretion of reactive oxygen species (such as H_2_O_2_), and reducing molecules (such as glutathione and GSH) (Chen et al., 2024). Together, these features create a highly dynamic and heterogeneous environment that promotes tumor cell proliferation, invasion, and metastasis. Overall, the dynamic changes in acidity, redox status, and specific metabolites of TME not only reflect the specific physiological characteristics of tumors, but also provide potential targets for the development of therapeutic strategies against TME.

#### 2.3.1 pH-responsive NDDS

Even when oxygen is plentiful, tumor cells tend to metabolize glucose through glycolysis rather than efficient oxidative phosphorylation. Glycolysis produces large amounts of lactic acid, resulting in a decrease in intracellular and extracellular pH (Warburg, 1925). At the same time, the enhancement of proton pump activity, abnormal blood vessels, overexpression of carbonic anhydrase, and regulation of hypoxia-inducing factors all work together to cause significant acidification of TME (Dorai et al., 2005).

Song has developed Cet-coupled DMAKO-20/PCL-PEOz micelles (DMAKO@PCL-PEOz-Cet) in which DMAKO-20 has been shown to have specific antitumor activity against CRC, but its hydrophobicity has been a puzzle (Song et al., 2024). Amphiphilic PCL-PEOZ solves this problem well and has been shown to promote the release of encapsulated drugs in acidic tumor environments due to the crystallinity of PCL and the pH sensitivity of PEOz. Similarly, Bu and Li et al. also demonstrated that using PCL-PEOz as a carrier to deliver DOX has a good cell-killing effect on tumor cells (Bu et al., 2019; Li et al., 2017).

Jia et al. developed a bifocal nano-delivery system (FCNP) based on DSPE-Hyd-mPEG for the co-delivery of fruquintinib (Fru) and siCCAT1 (Jia et al., 2023). The nanocomplex achieves specific accumulation at the tumor site through the EPR effect. Under acidic conditions in the tumor microenvironment, DSPE-Hyd-mPEG degrades in a pH response, resulting in the release of FCNP. Among them, Fru first acts on the tumor vascular system and exerts its rapid anti-angiogenesis effect. Subsequently, SICCAT1-loaded nanoparticles (CNP) were specifically taken up by tumor cells through receptor-mediated endocytosis, significantly improving the bioavailability of therapeutic molecules in tumor cells.

**FIGURE 4:**
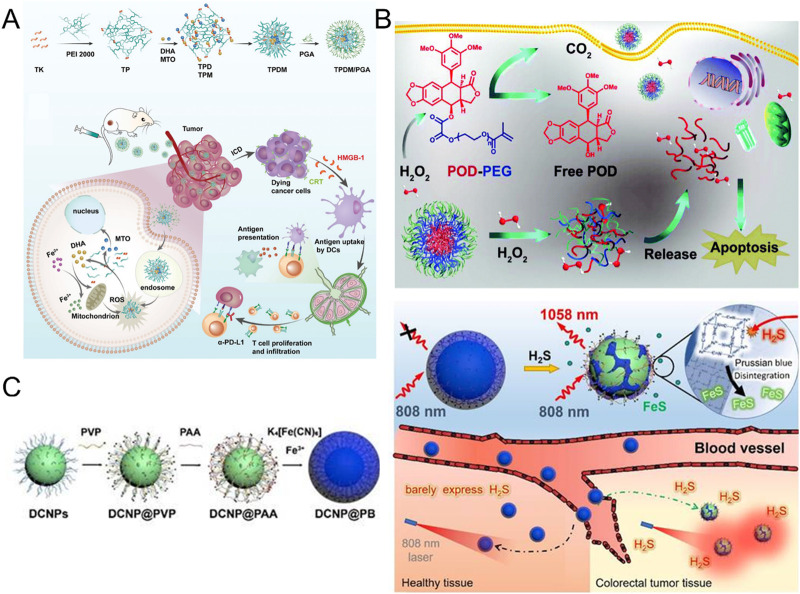
**(A)** Synthesis and tumor immunotherapy mechanisms of TPDM/PGA (Reproduced with permission from the reference: pH and ROS Dual-Responsive Autocatalytic Release System Potentiates Immunotherapy of Colorectal Cancer). Adapted from Su et al., Advanced Healthcare Materials, 2024, under a Creative Commons CC BY license. **(B)** Mechanism of action of H_2_O_2_-responsive POD-PEG NPs for cancer treatment (Reproduced with permission from the reference: H_2_O_2_-responsive nano-prodrug for podophyllotoxin delivery). Adapted from Ou et al., Biomaterials Science, 2019, under a Creative Commons CC BY license. **(C)** Preparation of DCNP@PB and the mechanism for the specific detection of H_2_S (Reproduced with permission from the reference: A H_2_S-activated NIR-II imaging probe for precise diagnosis and pathological evaluation of colorectal tumor). Adapted from Ji et al., Theranostics, 2025, under a Creative Commons CC BY license.

Malignant cells typically exhibit higher levels of reactive oxygen species (ROS) accumulation compared to normal cells. Based on this property, Su and his team developed an innovative pH and ROS dual-response drug delivery system (TPDM/PGA) (Su et al., 2024) (Figure 4A). The system specifically delivers DHA and MTO into tumor cells by the following mechanism: In the acidic TME, the carboxyl group in PGA undergoes deprotonation, resulting in the dissociation of PGA from the complex, exposing TPDM. Because TPDM is able to break down in response to the high concentration of ROS in cancer cells, DHA and MTO are released. These drugs further induce the production of more ROS, forming a positive feedback loop that accelerates drug release and exacerbates oxidative stress. This process not only enhances the immunogenicity of the tumor, but also significantly inhibits the growth of the tumor.

#### 2.3.2 GSH-responsive NDDS

GSH, as an important antioxidant, plays a key role in TME. Tumor cells promote the production and accumulation of GSH through the upregulation of key enzymes of GSH synthesis and activation of the Nrf2-Keap1 antioxidant signaling pathway (Zhang, 2010). In addition, hypoxia and acidic conditions also induce excessive secretion of GSH. On the one hand, high levels of GSH can neutralize ROS in tumor cells, protect tumor cells from oxidative damage, and inhibit anti-tumor immune response by regulating the function of immune cells (Niu et al., 2021). Therefore, the development of GSH consumables and GSH-responsive nanomedicines is a new direction in the treatment of tumors (Li Y. et al., 2023).

Li et al. developed an anticancer nano-delivery system Cu-Olsa@HA, and HA enhances its targeting and EPR effects (Li J. et al., 2022). In addition, the system takes advantage of the high concentration of GSH in the tumor microenvironment to achieve a valence transition between Cu^2+^ and Cu^+^. This transformation process not only effectively promotes the conversion of H_2_O_2_ to molecular oxygen O_2_, but also generates singlet oxygen ^1^O_2_ and hydroxyl radical OH with high cytotoxicity. This cascade eventually leads to the imbalance of redox homeostasis in tumor cells, thereby exerting its antitumor effect.

Yang et al. successfully reported a new nanoparticle pHCT74/MOF-5@DHA&CORM-401 in 2024. The nanoparticle design cleverly combines the specific targeting function of pHCT74 with the pH/GSH dual-responsive properties of metal-organic Framework 5 (MOF-5) (Yang et al., 2024a). pHCT74 precisely directs nanoparticle enrichment at tumor sites, while MOF-5 ensures the rapid release of active ingredients such as DHA and carbon monoxide-releasing molecule 401 (CORM-401) under acidic conditions in the tumor microenvironment. It not only promoted ROS-mediated ferroptosis and apoptosis, but also triggered immunogenic cell death (ICD).

#### 2.3.3 H_2_O_2_-responsive NDDS

In TME, the generation and metabolism imbalance of ROS is one of the important characteristics of tumorigenesis and development. H_2_O_2_, one of the main members of ROS, is often secreted in excess in the tumor microenvironment, and this phenomenon has been reported in multiple cancer types (Zaher et al., 2024). Excess H_2_O_2_ causes oxidative stress, DNA damage, protein oxidation, and lipid peroxidation, which induces apoptosis or necrosis (Rani et al., 2016). However, tumor cells often resist this oxidative damage by upregulating antioxidant defense mechanisms, thus surviving the harsh microenvironment. The excessive secretion of H_2_O_2_ in the tumor microenvironment is one of the important mechanisms for tumor cells to adapt to the harsh environment, promote their own growth, and evade immune surveillance (Wu, 2006). Therefore, nanomedicine targeting H_2_O_2_-responsive forms may become a new strategy for cancer treatment in the future.

Ou et al. used the H_2_O_2_-sensitive oxalate bond as the linking group to covalently link PEGMA to podophyllotoxin (POD) molecules and successfully constructed an amphiphilic prodrug molecule (POD-PEG) with H_2_O_2_ response characteristics (Ou et al., 2019) (Figure 4B). The prodrug molecules can form stable nanoparticles (POD-PEG-NPs) by self-assembly in aqueous solution. Benefit from EPR effect This NP accumulates specifically in tumor tissue. More importantly, based on the high H_2_O_2_ of TME, the oxalate bond in the prodrug molecule is broken, thus the POD molecule with anti-tumor activity is controllably released, and tumor-specific treatment is realized.

#### 2.3.4 H_2_S-responsive NDDS

In CRC, a significant physiological feature of TME is the high expression of endogenous hydrogen sulfide (H_2_S) (0.3–3.4 mM) (An et al., 2018). This phenomenon is mainly attributed to the selective upregulation of Cystathionine β-synthase (CBS) (Szabo et al., 2013). CBS is a key enzyme in the H_2_S biosynthetic pathway, and its overexpression leads to the accumulation of H_2_S in tumor tissues (Hellmich et al., 2015). This makes therapeutic intervention targeting the CBS-H_2_S axis likely to become an important direction of precision medicine in the future.

Chang et al. first used advanced nanomaterials synthesis technology to successfully prepare Cu_2_O nanoparticles with hollow mesoporous structures (Chang et al., 2020). A core-shell Cu_2_O@CaCO3 composite nanomaterial is formed by coating a CaCO_3_ shell uniformly on the Cu_2_O surface. Subsequently, HA was used for surface modification to enhance the tumor targeting of the material. The CaCO_3_ shell decomposes in the acidic microenvironment of tumor tissue, and Cu_2_O is converted to Cu_2-x_S with high activity under the action of endogenous H_2_S. This conversion not only gives the material excellent photothermal conversion performance, but also significantly enhances its photocatalytic activity, enabling efficient treatment of CRC.

Recently, Ji et al. made an important breakthrough in the field of accurate diagnosis and pathological evaluation of colorectal tumors (Ji et al., 2025) (Figure 4C). They successfully developed an H_2_S-activated NIR-II imaging probe consisting of Nd^3+^ doped down-conversion nanoparticles (DCNPs) coated with a Prussian blue (PB) layer, DCNP@PB. In this innovative design, PB not only acts as a response group for H_2_S, but also plays a key role in regulating the emission characteristics of DCNP@PB. Specifically, the coating of PB leads to a significant quenching of the luminescence of DCNP, which is mainly due to the overlapping effect between the absorption spectrum of PB and the excitation/emission spectrum of DCNP. However, when the probe arrived at the tumor site, Fe (III) in PB would react with the highly expressed H_2_S at the tumor site, thereby triggering the decomposition of the PB layer, resulting in a significant decrease in PB absorbance. This process allows the luminescence of DCNPs to be restored, thereby illuminating colorectal tumors with high sensitivity and specificity. It is particularly worth mentioning that the H_2_S response range of DCNP@PB is highly matched with the H_2_S concentration at the tumor site, which provides a strong guarantee for the specific detection of colorectal tumors in a complex physiological environment (Table 1).

**TABLE 1:** Advances of nano-drug delivery systems in colorectal cancer treatment.

Name	Size	Payload	Pathway/responsiveness	Study
E/AC-Au/MTX	∼390 nm	Methotrexate	Colonic pH	Bajracharya et al. (2023)
DAHBC NPs	∼60 nm	Curcumin	Colonic temperature and pH	Wang et al. (2019a)
NH_2_−MSNPs/CHT@EUS-100	∼100 nm	Catechin	Colonic pH	Kassem, et al. (2024)
5-FU ZGGO@SiO_2_@LRM	∼160 nm	5-FU	Colorectal targeting of bacterial membranes	Wang et al. (2019b)
BiNS@RHL-CS/5-FU	∼270 nm	5-FU, BiNS	β-glucosidase	Zhou et al. (2024)
encapsulated-DNPs	∼320 nm	Galunisertib	L1-CAM	Tramontano et al. (2022)
5-FU/CUR@SF	80–300 nm	5-FU, curcumin	EPR effect	Yuan et al. (2025)
HK NPs	∼100 nm	Honokiol	EPR effect	Ji et al. (2022)
ELR nanohybrids	∼50 nm	Docetaxel, Akt inhibitor	CD44 receptor	Gonzalez-Valdivieso et al. (2023)
SF–PLGA–PSAR–HA	∼240 nm	Sorafenib tosylate	CD44 receptor	Phatak et al. (2023)
HCNSP	∼50 nm	NLG919, PPa	CD44 receptor, GSH-responsiveness	Hu et al. (2020)
Cet-CINPs	70–150 nm	Irinotecan, cetuximab	EGFR receptor	Fang et al. (2023)
GCD Nanovehicles	∼10 nm	5-FU, miR-10b inhibitor	EGFR receptor	Wang et al. (2023a)
ICP@FA-PDA	∼100 nm	SN38, EGCG	Folate receptor	Zhang et al. (2025b)
Fe_3_O_4_@Au-DEX-CP-FA	∼60 nm	Cisplatin	EPR effect, Transferrin receptor	Alnasraui et al. (2023)
mHFn@MTO	∼20 nm	Mitoxantrone	Transferrin receptor	Cheng et al. (2024)
Tf@TBP-Ps-Dox	∼70 nm	Doxorubicin	Transferrin receptor	Wei et al. (2020)
SPIO-Tf	30–70 nm	—	Transferrin receptor	Zhang et al. (2025a)
RG@M-γ-CD CNPs	100–300 nm	Regorafenib	Mannose receptor	Bai et al. (2021)
Ce-G5-P-Ep	∼300 nm	Celastrol	EpCAM	Ge et al. (2020)
DMAKO@PCL-PEOz-Cet	∼120 nm	Cet, DMAKO-20	pH of TME	Song et al. (2024)
PEtOz-b-PU(SS)-b-PEtOz	∼150 nm	Doxorubicin	pH and reduction of TME	Bu et al. (2019)
PEtOz-SS-PCL43	∼100 nm	Doxorubicin	Reduction of TME	Li et al. (2017)
DSPE-Hyd-mPEG	∼60 nm	Fruquintinib, siCCAT1	pH of TME	Jia et al. (2023)
TPDM/PGA	∼180 nm	DHA, MTO	pH and ROS of TME	Su et al. (2024)
Cu-Olsa@HA	∼170 nm	—	GSH of TME	Li et al. (2022a)
POD-PEG-NPs	∼70 nm	Podophyllotoxin	H_2_O_2_ of TME	Ou et al. (2019)
Cu_2_O@CaCO_3_@HA	∼170 nm	—	pH and H_2_S of TME	Chang et al. (2020)
DCNP@PB	∼140 nm	—	H_2_S of TME	Ji et al. (2025)

## 3 Discussion

As a malignant tumor with high morbidity and mortality, the treatment strategy of CRC is undergoing a transformation from traditional chemotherapy and radiotherapy to targeted therapy and immunotherapy. In recent years, nanomedicine delivery systems, as an emerging therapeutic approach, have shown great potential in the field of CRC treatment. By encapsulating chemotherapeutic agents, targeted drugs, or immunomodulators in nanocarriers, NDDS can effectively improve drug targeting, minimize toxic side effects, enhance therapeutic efficacy, and overcome tumor resistance. Novel NDDS are also being progressively developed. Extracellular vesicles (EVs), as emerging nanomaterials, have garnered extensive attention. Endogenous EVs can reduce immune clearance, thereby demonstrating natural biocompatibility. Meanwhile, their high stability and affinity for tumor cells enable them to carry drugs to tumor sites for therapeutic effects (Yang et al., 2024b). Viral nanoparticles (VNPs) hold unique application prospects in gene therapy due to their efficient gene delivery capability and specific cell recognition. They can self-assemble into capsids to encapsulate therapeutic agents while retaining antigenic properties to induce immune responses, thus being utilized in vaccine development (Sun et al., 2024).

Although NDDS enhance drug efficacy through targeted delivery, microenvironment-responsive strategies, and multimodal synergies, their clinical translation remains challenging. The tumor microenvironment restricts precise drug delivery to all tumor regions, contributing to suboptimal therapeutic outcomes. While immune cells can target tumor cells, augmented immunosuppression weakens their infiltration and targeting capabilities. Additionally, due to limitations in preclinical research, insufficient comparative data on the efficacy and safety of different nanodrug types exist, making comprehensive evaluations unattainable. NDDS also face challenges in large-scale production due to inadequate production processes, difficult cost control, and susceptibility to stability issues during storage and transportation.

Therefore, based on this review, future NDDS development should prioritize safety and efficacy by developing advanced nanocarrier materials with enhanced biocompatibility, biodegradability, and targeting capability. Concurrently, tumor site-specific intelligent responsive, and dynamically regulated nano-systems must be designed to achieve precise drug release in the TME and minimize nonspecific toxic effects. Nano-drug design should be optimized through surface modification and drug co-loading strategies to explore synergistic mechanisms with immune checkpoint inhibitors, oncolytic viruses, etc., thereby further enhancing targeting specificity and therapeutic efficacy. In-depth investigations into NDDS-TME interactions are critical to elucidate distribution, metabolism, and clearance mechanisms within tumor tissues, providing a theoretical foundation for optimizing nanodrug design. Personalized nano-drug combinations should be customized based on CRC molecular typing and gut microbiota characteristics to achieve precision therapy. Simultaneously, innovative production processes are needed to reduce costs and improve batch consistency. Strengthening the interface between basic research and clinical translation will facilitate the transition of nanodrugs from bench to bedside. In summary, NDDS represent a promising strategy for CRC treatment. With continuous advancements in nanotechnology, NDDS are poised to play an increasingly pivotal role in improving therapeutic outcomes for colorectal cancer patients.
